# GOF Mutant p53 in Cancers: A Therapeutic Challenge

**DOI:** 10.3390/cancers14205091

**Published:** 2022-10-18

**Authors:** Lobsang Dolma, Patricia A. J. Muller

**Affiliations:** 1 CRUK Manchester, University of Manchester, Alderley Park, Manchester SK10 4TG, UK; 2 Department of Biosciences, Durham University, Stockton Road, Durham DH1 3LE, UK

**Keywords:** mutant p53 chemoresistance, targeted therapy, gain-of-function, GOF

## Abstract

**Simple Summary:**

In normal cells, p53 is a protein which regulates the cell cycle progression to ensure normal cell division, growth, and development. However, in cancer, changes in the p53 DNA sequence, called genetic mutation, results in the protein either losing its normal function or exhibiting advanced pro-tumorigenic functions that lead to cancer. Importantly, cancers with mutations in the p53 protein often represent ones which are more aggressive and more resistant to chemotherapy. As a result, many studies have and continue to investigate multiple ways to target mutant p53-bearing cancer using targeted therapy, gene therapy, immunotherapy, and combination therapies. Knowledge of these strategies is important in improving the overall therapeutic response of cancers with mutant p53. This review highlights new strategies and discusses the progression of such therapies.

**Abstract:**

*TP53* is mutated in the majority of human cancers. Mutations can lead to loss of p53 expression or expression of mutant versions of the p53 protein. These mutant p53 proteins have oncogenic potential. They can inhibit any remaining WTp53 in a dominant negative manner, or they can acquire new functions that promote tumour growth, invasion, metastasis and chemoresistance. In this review we explore some of the mechanisms that make mutant p53 cells resistant to chemotherapy. As mutant p53 tumours are resistant to many traditional chemotherapies, many have sought to explore new ways of targeting mutant p53 tumours and reinstate chemosensitivity. These approaches include targeting of mutant p53 stability, mutant p53 binding partners and downstream pathways, p53 vaccines, restoration of WTp53 function, and WTp53 gene delivery. The current advances and challenges of these strategies are discussed.

## 1. p53 and Mutations in Cancers

p53 is a tumour suppressor protein and nuclear transcription factor (53 kDa) regulating target genes and involved in apoptosis, senescence, cell cycle arrest, and DNA repair. In response to low doses of genomic stress, both extrinsic (e.g., UV-induced DNA damage) and intrinsic (e.g., chromosomal aberrations), p53 regulates cell cycle arrest to allow for DNA repair [1,2,3]. In response to high doses of stress, p53 is more likely to promote apoptosis. Importantly, many chemotherapeutics act by inducing this stress-induced cell death function of p53 to destroy tumour cells.

**Figure 1:**
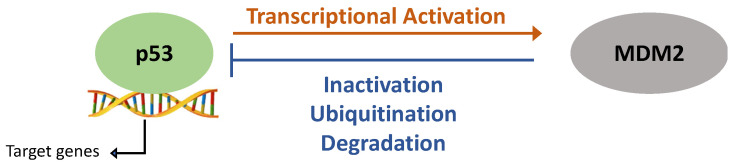
p53 and mouse double minute-2 (MDM2) auto-feedback loop. DNA damage and cellular stress increase p53 expression and facilitate its nuclear import. This allows for p53’s transcriptional activation of target genes, including MDM2. p53-induced MDM2 activation then results in p53 binding to MDM2 and its proteasomal degradation.

In the absence of stress, p53 protein expression is kept at low levels [4]. This is facilitated by the E3 ubiquitin ligase MDM-2 (mouse double minute-2) that ubiquitinates p53 leading to its degradation. In response to DNA damage, p53 is released from MDM2 suppression allowing for p53-mediated transcription. MDM-2 limits p53 expression whilst p53 directly promotes MDM-2 expression. This creates an autofeedback loop that allows for a fast and dynamic signalling response to react to differences in stress quickly (Figure 1) [5,6].

*TP53* mutation occurs in ~50–60% of all human cancers and can result in both the absence of protein expression or the expression of a mutated protein [7]. p53 mutational status within tumours is heterogeneous and the onset of *TP53* mutations can vary greatly in different cancers. As an example, in colorectal [8], breast [9], and pancreatic [10] cancers, *TP53* mutation is marked as a late stage tumourigenic event aiding more with tumour progression than with tumour initiation, while in pre-malignant breast lesions [11], hepatocellular carcinoma [12], and in astrocytoma [13] *TP53* mutations present during the early stages of tumorigenesis. 

**Figure 2:**
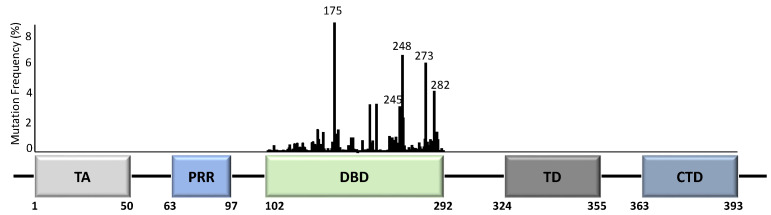
TP53 structure and mutation distribution (%) within the DNA-binding domain (DBD). The frequency of each mutation in all cancers based on the p53 database (www.p53.fr) is indicated for the DBD of TP53. Amino acid positions are indicated below the domains. Five TP53 hotspot mutation sites are further indicated with codon numbers above the bars. TA = transactivation domain; PRR = proline-rich region; DBD = DNA-binding domain; TD = tetramerization domain; CTD = carboxyl terminal regulatory domain.

Unlike most other tumour suppressor genes, *TP53* mutations often affect a single allele with loss of expression from the remaining allele [14]. This occurs via deletion of part of chromosome 17p [15], methylation of the second allele [16], or through additional mutations [17]. Principally, whilst the presence of *TP53* mutations span across almost all of its 393 aa residues (Figure 2), the specificity and frequency of the >25,000 registered *TP53* mutations can be differential based on the tumour type, with individual mutants often showing different phenotypical changes [18,19]. Importantly, most mutations are found in the DNA-binding domain (DBD) with six hotspot mutations at codons 175, 245, 248, 249, 273, and 282 (Figure 2) [14,20].

*TP53* mutations can cause truncations or frameshifts in *TP53* that almost always result in loss of p53 expression. Missense mutations generally result in expression of mutant proteins with one amino acid variation from WTp53 [14,18]. This generates a stable mutant p53 protein with longer half-life, seen as increased expression in human cancers [21]. These mutant proteins, including all hotspots, can have alterations in the protein’s structure such as unfolding of the DBD (conformational/structural mutants) [22] or a decreased DNA binding ability (contact mutants) [18].

Mutant p53 proteins often lose some or all of p53’s tumour suppressive function (loss-of-function, LOF) but may also acquire gain-of-function (GOF). This GOF resembles an oncogenic phenotype and is independent of WTp53 [18]. We and others have shown that mutant p53 promotes invasion and metastasis, tumour growth, genomic instability and chemoresistance [23,24,25,26], via a multitude of different mechanisms (reviewed in [27,28,29,30]). Mutant p53 proteins can further have dominant-negative effects over the remaining WT protein [20,28]. This was attributed to mutp53’s ability to form hetero–tetramer complexes with the WTp53 protein [20], causing multimer inactivation [31]. This was seen for both contact and conformational p53 mutants [20]. 

## 2. Mutant p53 and Chemoresistance

Mutp53 forms a challenging anti-cancer therapeutic target, mainly due to its lack of druggable allosteric sites, the occurrence of thermodynamically disrupted states as well as its intrinsic ability to confer drug resistance [24,32,33].

**Figure 3:**
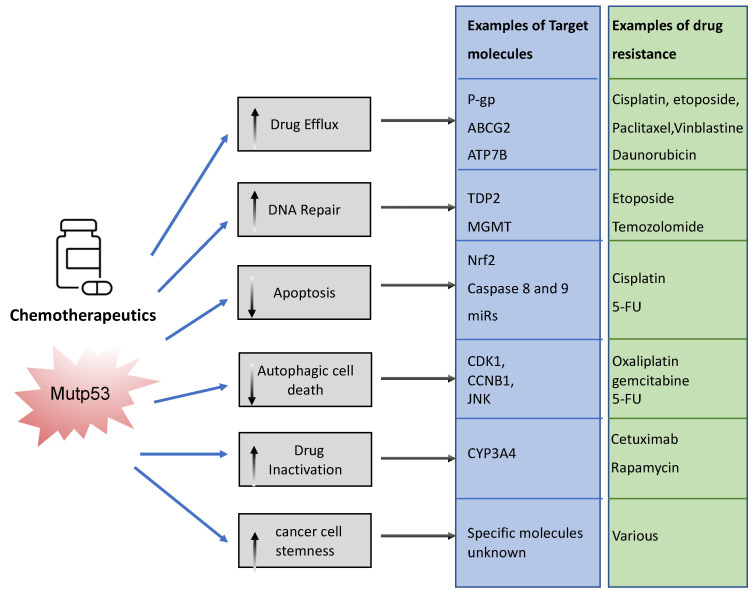
Examples of ways in which mutant p53 promotes chemoresistance. Mutant p53 can impact various cellular processes to prevent chemotherapeutics drugs from working. It can neutralize chemotherapy, promote efflux and impair the way in which these drugs promote cancer cell death. This figure shows examples of these pathways.

The association between mutp53 expression and decreased chemosensitivity is seen in various primary cancers, including breast [34], ovarian [35], lung [36], and hematopoietic [37]. Loss of WTp53 expression can underlie this chemoresistance, but there are also ways in which mutant p53 acquires chemoresistance via its GOF [33]. Mechanisms include, but are not limited to, upregulation of drug transporters, enhanced DNA repair, activation of stemness, apoptosis avoidance, and drug inactivation (Figure 3). 

To limit toxicity of drugs, mutp53 can directly act on drug availability by regulating drug efflux or drug stability. Mutp53 promotes expression of the (*MDR1*) gene encoding for the ATP-binding cassette (ABC) transmembrane transporter ABCB1/P-glycoprotein (P-gp) [38]. P-gp extrudes xenobiotic substances/toxic compounds and chemotherapeutic drugs [39]. The transcriptional upregulation of P-gp in cancer is driven by GOF-mutp53’s direct binding to *MDR1′s* promoter [40]. This enhances drug efflux, reduces drug absorption, and minimizes drug retention/accumulation, causing resistance to anti-cancer drugs, such as taxanes (paclitaxel), vinca alkaloids (vinblastine), and anthracyclines (daunorubicin) [41]. Of note, other ABC transporters such as ABCG2 can also be upregulated by mutant p53 to enhance secretion of 5-flouracil (5-FU) [42]. Interestingly, mutant p53 does not only regulate the gene expression of such transporters. We recently discovered that mutp53 also specifically enhances plasma membrane expression of P-gp and ATP7B in response to cisplatin and etoposide to enhance efflux, perhaps working in concert with transcriptional regulation of such transporters [24,26]. GOF-mutp53 (R248W and R282W) can also directly inactivate chemotoxic drugs by upregulating cytochrome P450 enzyme 3A4 (CYP3A4) that help neutralize these drugs [43]. 

In order to limit toxicity of drugs, mutp53 intercepts in many downstream signalling pathways. In response to drug-induced DNA damage, mutant p53 promotes DNA repair. As an example, GOF-mutp53 (R175H and R248Q) promoted etoposide resistance by enhancing tyrosyl-DNA phosphodiesterase 2′s (TDP2) expression in lung cancer cells in an Ets2 dependent manner [44]. TDP2 in turn repaired etoposide induced double-strand DNA breaks [45], resulting in chemoresistance. Likewise, GOF-mutp53 upregulated the expression of O(6)-methylguanine-DNA-methyltransferase (MGMT) in glioblastoma, enabling the repair of alkylation induced DNA damage by temozolomide [46]. Mutp53 can also directly or indirectly prevent apoptosis. Transcriptionally, it can upregulate Nrf2 (nuclear factor erythroid 2-related factor 2) in response to cisplatin to induce expression of the anti-apoptotic mitochondrial genes: *Bcl2* and *Bcl-xL* [47]. Alternatively, GOF-mutp53’s apoptotic resistance can also occur via direct inhibition of caspases 8 and 9 [48,49,50] or through transcriptional upregulation of miRs that target the apoptosis machinery [51]. Many of the chemotherapeutics are known to cause autophagic cell death through apoptosis. Mutp53 can avoid apoptosis by inducing autophagy via the mTor/AMPK signalling pathway [52], although autophagy itself also regulates mutp53 expression (see Section 3.1.2).

It is likely that in an actual cancer, mutant p53 employs one or more of these mechanisms to combat chemotherapeutics, resulting in selection for p53 mutations. In fact, selection of mutp53 is driven by the fact that mutp53 actively promotes stemness [53]. This could be seen as promoting chemoresistance because cancer stem cells are relatively quiescent and therefore less vulnerable to chemotherapy that predominantly acts on highly proliferative cells [53].

## 3. Strategies That Exploit Mutant p53 Expression

Numerous current and previous studies have explored targeted treatment strategies that either directly target mutp53 or exploit the cancers’ dependence on pathways that rely on mutp53 expression [54]. However, so far, very few of these have progressed to pre-clinical/clinical trial studies and many have resorted to trialling combinations of various treatments. 

**Figure 4:**
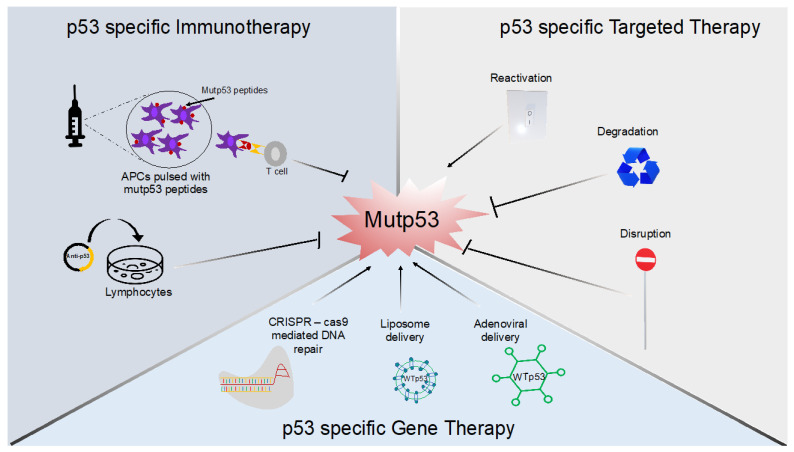
Mutant p53-targeting therapies. Examples of three treatment strategies specifically targeting mutant p53 using immunotherapy, targeted therapy, or gene therapy are shown.

In this review, we provide an updated review on potential therapeutic strategies, both current and new, that can be employed in mutp53-bearing cancers. The reviewed strategies are grouped under the three main types of p53 specific anti-cancer treatment approaches: mutant p53-targeted therapy, gene delivery therapy, and immunotherapy (Figure 4). We also discuss the option to combine these therapies. 

### 3.1. Mutant p53-Specific Targeted Therapy

Most mutant p53 targeting strategies in cancers have focused on incorporating one of three key mechanisms: reactivation of mutp53 into a WTp53-like state [55,56,57], degradation of mutp53 [58,59,60], or perturbation of mutp53’s function while reactivating WT function [61]. These mechanisms are appealing because expression of mutant p53 in cancers is often high, whereas WTp53 expression in normal tissue is low [62], allowing for specific targeting of mutant p53 with minimal side effects.

#### 3.1.1. Reactivating Mutant p53 to Behave like a WT Molecule

Most common p53 missense mutations, including all hotspots have a complete or partial loss of WT function [63,64]. Many mutants exist in a distorted conformation that prevents them from binding to the DNA and exert p53 function. The p53 hotspot mutant R175H, a conformational mutant, has been most extensively studied. Murine studies showed that expressing WTp53 or restoring WTp53 expression causes tumour regression [56], tumour clearance [57], senescence [55,57] and increased survival [56], suggesting that reactivation therapies in which mutants are converted to a WT molecule could work in vivo. 

The earliest discovery of a small molecule compound that was reported to mediate mutp53 reactivation was CP31398 [65,66]. The compound was initially suggested to bind to mutp53’s core domain [66], although a later study contested this binding and instead suggested the compound functioned as a DNA intercalating agent that exhibits both p53-dependent and -independent anti-tumour activity [67]. Subsequent studies then discovered molecules such as PRIMA-1, APR-246, MIRA-1, and STIMA-1, which are called Michael acceptors due to their ability to bind covalently with p53’s cysteine residues [68,69]. This results in an enhanced thermal stability of reactivated mutp53 in a WTp53-like folding state [69] and tumour growth inhibition [65,70,71]. APR-246, the methylated form of PRIMA-1 is one of the few p53 specific targeted therapies which has progressed to clinical trials [65,70,72]. Another p53-specific targeted therapy in clinical trials is COTI-2, a thiosemicarbazone compound, which primarily induces Zn^+2^ chelation-mediated p53 refolding, restoring p53’s DNA binding capacity [73,74]. However, the molecule’s pro-apoptotic effect was reported to be both p53-dependent and -independent in pre-clinical models [73,75]. More recently, arsenic trioxide, a cysteine reactive compound stabilizes the DNA-binding loop–sheet–helix motif alongside the overall β-sandwich fold of p53 structural mutants through covalent binding [76]. This drug restored p53’s transcriptional activity both in vitro and in vivo and is currently in clinical trial for patients with AML (acute myeloid leukaemia) [76,77].

Compounds that target specific p53 mutants using covalent binding have also been developed. PK083 and PK7088 were synthesized to target the base substitution-induced cavity in hotspot Y220C p53 mutants [71]. In particular, PK7088 was found to promote re-folding of the Y220C p53 mutant with subsequent induction of p53 target genes’ (p21 and NOXA) expression [71]. 

Compounds such as SCH529074 [78] and peptides like CDB3 [79] were developed to act as chaperones by non-covalently binding to mutp53’s DBD and consequently restoring WTp53 activity. Interestingly, a similar non-covalent binding could also be demonstrated by using peptides such as pCAPs, that change the equilibrium between unfolded and folded p53 states with their stronger binding affinity (non-covalent) to WTp53 over mutp53 (R175H and R273H) [80]. 

The interest of researchers in almost all of these drugs originates from the notion that mutp53 expression in cancers is so high that, upon conversion into a WTp53-like molecule, a death response is likely initiated, making such compounds likely to act even in the absence of additional therapies. However, they are generally explored in conjunction with conventional chemotherapy to further enhance WTp53-dependent cell death. Although these compounds were designed to target structural mutants, DNA contact mutants as well as WTp53 can under certain conditions unfold (e.g., in hypoxia) [81]. This would suggest that these compounds could target other mutants, dependent on the tumour environment. 

In many tumours, instead of missense mutations, p53 expression is lost due to nonsense TP53 mutations, such as Q192X and E298X, resulting in truncated p53 mRNA expression, nonsense mediated mRNA decay (NMD), and subsequent loss of protein expression. Compounds like Ataluren [82] and aminoglycoside Geneticin (G418) [83] were reported to induce enhanced translational readthrough of the p53 mutants, resulting in the translation of full-length p53 protein that was functionally active. However, the potential high cytotoxicity of read-through-inducing treatments such as G418 remains a major challenge for long-term clinical application [84].

#### 3.1.2. Induction of Mutp53 Degradation

Although counterintuitive at first glance, mutant p53 degradation strategies are also explored as a mutp53 targeting strategy in the clinic [60,77,85,86,87,88,89]. The idea behind removal strategies is that mutp53 causes genetic changes in the cancer cells that make them dependent on mutp53 expression [90]. Many cell lines in which mutp53 expression is severely reduced are impaired in growth and do not survive when xenografted in mice [91,92]. Importantly, loss of mutp53 expression in vivo also resulted in decreased tumour growth and tumour regression [93,94].

As the use of CRISPR or RNAi in the clinic is still mainly exploratory, many researchers have focused on decreasing mutp53 stability with drugs. In particular, the inhibition of Heat-shock protein 90 (Hsp90) and Histone deacetylase 6 (HDAC6) chaperone complex which stabilizes mutp53 remains the most studied method for mutp53 degradation. Mechanistically, the Hsp90 protein conceals the ARF-binding site of MDM2 protein, preventing p53 degradation [58]. The earliest use of an Hsp90 inhibitor, Geldanamycin, in mutp53 cancer cell lines reduced mutant p53 expression and concomitantly refolded mutp53 into a more WT-like conformation [95]. Similarly, Hsp90 inhibition by 17AAG (17-allylamino-17-demethoxygeldanamycin) activated MDM-2 and another mutp53 targeting E3 ubiquitin ligase, CHIP (carboxy-terminus of Hsp70-interacting protein), to degrade mutp53 [60]. Notably, Ganetespib, a more potent Hsp90 inhibitor was in a Phase III clinical trial GALAXY-2 for patients with advanced NSCLC, but has been terminated early due to lack of significant improvement in overall survival [96]. 

HDAC6 is believed to activate Hsp90 and promote Hsp90′s inhibition of MDM2 and CHIP [97] and is therefore also of interest to destabilize mutp53. Indeed, inhibitors such as FR901228, the FDA approved drug SAHA [59], A542 and trichostatin A caused Hsp90-dependent mutp53 depletion [98]. In contrast to proteasomal degradation, SAHA was shown to cause mutp53 degradation through autophagy [99]. Spautin-1′s inhibition of macro autophagy and consequent activation of chaperone-mediated autophagy (CMA) caused lysosomal uptake and degradation of mutp53 upon nutrient deprivation [100]. 

In 2012, Freed-Pastor et al. reported that GOF-mutp53 regulated the mevalonate pathway to promote tumorigenesis [101]. Statins inhibit this pathway and were studied as agents that block mutp53 downstream signalling. More recently, statins were also found to destabilize mutant p53 through DNAJA1 and CHIP-mediated ubiquitination [102]. A retrospective study investigating statin use in lung cancer, found that the usage of statins reduced the 5-year mortality [103]. It would be interesting to see if this changes dependent on p53 status in these cancers.

Numerous other compounds promote degradation of mutp53, including, but not limited to, disulfiram [104], YK-3-237 [105], arsenic trioxide [106], NSC59984 [107], and gold nanoparticles [108], although the specificity, the exact mechanism of degradation and/or the clinical use of such compounds needs further investigation. 

#### 3.1.3. Disruption of Mutp53 Function

Mutp53 function often relies on binding partner proteins. These include other transcription factors, the p53 family members p63 and p73, ETS1, SREBP, but also other proteins such as Pin1 [109]. Binding of mutp53 to these proteins either inhibits their function (e.g., p53 family members) or potentiates their function (e.g., ETS1). Mutp53 treatment strategies aim to prevent these interactions or disturb downstream signalling. 

p63 and p73 are p53 homologs and exist in different isoforms in various tissues and tumours [110,111]. In humans, p63 is important for embryonic development, differentiation and for epithelial cell maintenance [112,113,114,115]. Likewise, p73 regulates cytoskeletal rearrangement [116], cell adhesion [117,118], ciliogenesis [119], and planar cell polarity [120]. The full-length versions of these proteins, TAp63 and TAp73, are generally thought to have tumour suppressive function [110,111]. Mutp53 proteins inhibit TAp63 and TAp73 function and so promote tumorigenesis, metastasis, and chemoresistance [14]. Most strategies focus on disruption of mutp53’s inhibitory interaction with p73 [121]. Prodigiosin facilitated p73 upregulation by disrupting mutp53’s interaction with p73. This induced WTp53-like transcriptional activity of p73 with p21 activation and anti-tumour potential [122]. Compounds such as RETRA [61] and short interfering mutant p53 peptides (SIMPs) [123], also act by disrupting mutp53’s interaction with p73 and restoring its function. Interestingly, a compound called 1-carbaldehyde-3,4-dimethoxyxanthone (LEM2) was found to prevent mutp53’s inhibition of TAp73*α* (a C-terminal splice variant with tumour suppressive function) by disrupting both mutp53 and MDM2 binding to p73 in neuroblastoma, further enhancing p73 function [124]. 

The above approaches are only a handful of approaches that are currently being explored to tackle mutp53 cancers. Other approaches target mutp53 downstream signalling pathways including the EGFR signalling pathway with drugs such as NA20 [125] or cetuximab [126] use synthetic lethal approaches to find vulnerabilities of mutant p53 cancer cells that can be targeted with drugs [127,128] or target the capacity of mutp53 to form aggregates using compounds such as ReAcp53 [129,130].

Importantly, novel discoveries on the function and consequence of mutp53 expression in cancers are still being made on a fairly regular basis. It is likely that effective strategies that disrupt mutp53 function rely on a much better understanding of the mechanisms underlying all of mutp53’s actions. 

### 3.2. Mutant p53-Specific Gene Therapy

Re-expressing p53 using p53 gene therapy is a very appealing strategy to allow for restoration of a p53-mediated cell-death response upon chemotherapeutic challenge in cancer cells. However, restoring p53 expression in tumours remains challenging and has mainly been approached using viral delivery or nanotherapeutics/lipid particle delivery of p53. 

p53 viral gene delivery research started around 1994 and used replication-deficient recombinant adenovirus in tumour cell lines, in xenografts, and in orthotopic murine models. In all cases, a p53-dependent growth inhibition and marked apoptotic response could be detected when viruses successfully delivered p53 to the target cells [55,57,131,132,133,134]. 

The Onyx company developed a tumour-restricted adenovirus for WTp53 gene delivery and reported effective replication of the virus in cells with p53 mutants but not WTp53 cells [135]. Likewise, in China, Gendicine is an approved recombinant p53 adenovirus gene therapy product that was initially administered in combination with radiotherapy to treat head and neck cancer, but it has now also been reported effective in other cancers [136].

A major limitation of replication-deficient viruses is efficiency with not all tumour cells being targeted and effects likely to be transient [133]. Frequent re-dosing to ensure a long-term effect might therefore be necessary. Replication competent viruses or oncolytic viruses, known as CRAdp53 vectors [137], including ONYX15 [138], SG600-p53 [139], AdDelta24-p53 [137], and H101 [140] were therefore developed and could negate some of these problems, although the safety of using such viruses needs further testing. Interestingly, even though some of the p53 gene therapy’s WT-p53 protein expression in only some cells, secondary effects in inducing systemic immunological response led to long-lasting effects on tumour regression [55,57]. In a hepatocarcinoma mouse model, re-expression of p53 using a doxycycline model induced a cooperative mechanism between tumour cell senescence and the innate immune system leading to complete tumour regression [57]. These data suggest that expression of p53 in only part of the tumour cells might be sufficient to trigger an immune response to eliminate more than just the infected cells.

As an alternative to viral delivery, liposome-mediated delivery of WTp53 protein was studied in head and neck cancer [141]. By targeting the liposomes with transferrin (a ligand recognized by the transferrin receptor that is expressed to high levels on cancer cells), it was possible to deliver WTp53 and cause tumour regression [141]. This strategy was further developed into a clinical nano-therapy treatment, SGT-53, that is currently in advanced clinical trial stage for various solid cancers and even for COVID-19, in which p53 is thought to play a role in viral infection [142,143,144]. 

p53 restoration gene therapy has mostly been studied in tumours without p53. It seems plausible that the potential ability of GOF-p53 mutants to induce a dominant negative effect on reintroduced WTp53 would negate the effect of the p53 gene therapy. However, in one of the earliest studies, the dominant negative p53 mutants did not abrogate adenoviral transmitted WTp53 protein function [132]. Some reports suggest that the ONYX-15 p53 gene therapy actually relies on deregulated p53 signalling and works best in mutp53 cells [145], although others demonstrated enhanced oncolytic activity of ONYX-15 in WTp53 cells [146] or activity that was independent on p53 status [147,148]. More recently, parvovirus targeting of glioblastoma stem cells with a cytotoxic protein NS1 showed that this virus is more effective in mutp53 cells [149]. These studies likely suggest disease/cell specificity and raise the question on how p53 is involved in viral infection, viral replication, and which patient would benefit most from such therapies. 

Although technically not a p53 gene delivery strategy, a more recently CRISPR/Cas9-based therapeutic vector (inducible and tumour specific) has been proposed to restore p53 function in mutp53 cells [150,151]. This technique could replace the mutated p53 locus with a functional p53 copy through homologous recombination. Just like gene delivery, delivery of the CRISPR/Cas9 system could be done via viral vectors or lipid particles, although functionality still needs to be demonstrated in cells [150,151]. Notably, CRISPR/Cas has recently been shown to select for p53 mutations in p53 WT cells, which could indicate that there are restrictions associated with this approach [152]. 

In conclusion, p53 gene delivery could be an effective strategy as long as the hurdles to effective delivery, side-effects, and selectivity can be addressed in the future.

### 3.3. Mutant p53 Specific Immunotherapy

Immunotherapy is one of the most prominent methods that can provide long-term tumour regression [153], as has been demonstrated with therapy targeted against K-RAS and other mutations [153,154]. In contrast to K-RAS, p53 is mutated on many more amino acids, making it challenging to find a specific ‘mutp53’ targeting region for immunotherapy. Preliminary studies and trials have shown promising data corresponding to the immunogenicity of mutp53-derived peptide epitopes. These peptides were used to generate activated T-cell response in vitro for in vivo delivery via vaccination [155,156,157,158]. Likewise, a high-throughput screening of p53 hotspot mutants (R175H, Y220C, G245S, R248Q, R248W, and R282W) found neoantigens in metastatic epithelial cancers. A mutp53-specific T-cell response was elicited when co-cultured with autologous APCs (antigen-presenting cells) that then recognized mutant p53 [153]. Of note, cells with different p53 mutants had different capacities to present immunogenic epitopes [153], suggesting a potential link between the types of p53 mutants and their immunogenicity in different cancers. 

As WTp53 is expressed at low levels in normal cells and mutp53 accumulates in tumour cells, immunotherapy using WTp53 peptide has also been explored [159]. The efficacy of this approach is supported by the observation that a WTp53 peptide induced a p53-specific cytotoxic T-cell response against mutp53/WTp53 in both mice [160,161] and in cancer patients [159,162,163,164]. One study also showed selective killing of tumour cells over normal cells [159]. Importantly, this method would bypass the requirement of mutp53-specific immunogenicity. 

Over 20 different clinical trials have been conducted using p53 vaccination as a strategy to combat cancers. Although a p53 response is seen and vaccines are generally considered safe, a phase II trial did not show enough benefit to warrant progression to phase III trial [165]. Therefore, further research is needed to enhance immune strategies for mutant p53 in the future. 

### 3.4. Mutant p53 Specific Combination Therapy

For future anti-cancer therapeutic application, the effect of a single strategy application might not be sufficient to cause long-term tumoricidal effects or to overcome potential resistance. This could be resolved by combining the different methods discussed above or by coupling of p53 specific therapeutic strategies with other therapies that target relevant pathways to exploit synergistic effects for improved therapeutic benefit. 

Past and ongoing trials like to combine p53 immunotherapy with p53 gene therapy. As an example, one strategy includes priming the autologous lymphocytes with anti-p53 genes in vitro, followed by their in administration in patients [166]. The use of this strategy has rendered promising therapeutic potential in xenograft studies [167]. However, the clinical application of this strategy in humans, so far, has not shown evidences of objective tumour response [167] due to lack of p53-specific self-tolerance and the presence of immunogenicity against the viral vectors. 

Another strategy combines p53-specific gene therapy with conventional immunotherapy by direct administration of adenoviral p53 (Ad-p53) gene therapy with immune checkpoint inhibitors such as anti-PD1 or agonists of cytokines such as CD122. This is to stimulate a WT-p53 specific immune response using the Ad-p53 and at the same time, bypass the immune checkpoint blockade normally found in cancer with inhibitors such as anti-PD1. This strategy has been investigated in recurrent and metastatic cancers [168] and was reported to cause effective tumour remission in murine tumour models [169]. 

p53-specific gene or immunotherapy with conventional chemotherapy in more advanced and often chemo-resistant cancers has also been explored. As an example, in platinum resistant ovarian cancer patients (phase I), the combined therapy of modified vaccinia Ankara vaccine delivering wild-type human p53 (p53MVA) with gemcitabine chemotherapy yielded immunological response in some patients with durable disease control [170]. This study also proposed that the combination therapy of p53MVA with immunotherapeutic agents that have immunomodulatory effects, such as anti-CTLA-4, would improve therapeutic effect [170]. 

Numerous combinations of p53-specific targeted therapies have been trialled with conventional chemotherapies or cancer-specific treatments in different types of cancers. In particular, APR246 has been shown to synergize with DNA-damaging anti-cancer drugs cisplatin and adriamycin [171,172]. This was attributed to the synergistic crosstalk between APR-246′s reactivation of mutp53 sensitizing the tumour cells to the DNA damaging agents [65]. APR-246 was also noted to demonstrate enhanced synergy with inhibitors of other cancer signalling, including proteosome inhibitor carfilzomib [173], BRAF inhibitor vemurafenib [174], Poly (ADP-ribose) polymerase (PARP) inhibitor Olaparib [175], etc. Other mutant p53 reactivating strategies also show promise for combination therapy. As an example, a phase II clinical trial for myelodysplastic syndrome (MDS)/acute myeloid leukaemia (AML) patients is currently being conducted combining arsenic trioxide with decitabine and cytarabine to treat MDS and AML, respectively [77]. 

Finally, completely novel vulnerabilities created by mutant p53 expression are explored for synthetic lethality. An example of this is the acetylation of codon 158 in mutp53 cancers. Acetylation of this mutant by a variety of acetylators, including HDAC, JQ1, and topotecan makes this mutant vulnerable to cisplatin-induced cell death [176].

Taken together, it is likely that given the role of p53 and the mutant form of p53 in many different cell processes, not one single therapy will be totally successful in eliminating all mutp53-bearing tumour cells. Combining current chemotherapy with new therapy is often the way in which new drugs are trialled in cancer and therefore would form the easiest and most cost-effective combination to explore. 

## 4. Conclusions and Future Perspectives

The search for mutp53-specific treatment strategies began almost two decades ago. However, very few have advanced to clinical trial stages, with none being currently approved for patient treatments (except for Gendicine in China). This is in part caused by the elusiveness of p53 mutants existing in different states/forms (e.g., conformational or contact mutants) bearing different targetabilities as well as the intrinsic ability of mutp53 to overcome its dependence on multiple pathways and allow for resistance to various treatment regimes. 

However, with p53-specific targeted therapy, the development of small molecules that directly or indirectly target mutp53 has proven to be highly promising particularly with discoveries such as APR-246 and COTI-2, currently in clinical trials. Likewise, targeted inhibition of mutp53’s molecular chaperones such as HDAC-6 and hsp90 has progressed to pre-clinical and phase I, II and III clinical trial studies, respectively. Yet, the key challenge involving p53-specific targeted therapy remains selectivity and specificity of the compound ensuring the delivery works in the targeted cells with minimal effect on normal cells.

For p53-specific immunotherapy, the data shown so far reflect the important knowledge that both mutp53 and WTp53 are immunogenic and can elicit tumoricidal immunogenic reactions from immune cells in vivo. However, whilst adoptive cell therapy against mutp53 appears to be promising, it potentially could facilitate a more effective and durable objective clinical response when combined sequentially with conventional chemotherapy or simultaneously with gene therapy. 

p53-specific gene delivery therapy has a highly encouraging therapeutic potential but requires further optimization to improve efficacy and reduce toxicity. Long-term validation within in vivo models with delivery systems capable of ensuring uniform systemic transfections/infections will be pivotal. More importantly, the combination of p53-specific gene therapy in the form of p53 cancer vaccines with targeted immunotherapy could be one of the more important strategies that bears significant potential and necessitates further investigations.
